# Notes on the Additions Made to the British Pharmacopœia, 1890

**Published:** 1891-09

**Authors:** 


*Notes on the Additions made to the British Pharmacopœia,* 1890. By FREDERICK T. ROBERTS, M.D., B.Sc., F.R.C.P. Pp. 22. London: H. K. LEWIS. 1891.

*New Official Remedies, British Pharmacopœia,* 1890. *Supplement to "Materia Medica and Pharmacy."* By A. W. GERRARD. Pp. 40. London: H. K. LEWIS. 1891.

These little books were rendered necessary by the issue of the *Additions to the British Pharmacopœia,* 1890: they form supplements to the well-known works of their respective authors; and the arrangement of the matter in each follows the method adopted in the larger book. They both seem very well adapted to the needs of students. The second has the advantage of an index.

